# cDNA sequencing increases the molecular diagnostic yield in Chediak-Higashi syndrome

**DOI:** 10.3389/fgene.2023.1072784

**Published:** 2023-03-08

**Authors:** Chulaluk Kuptanon, Marie Morimoto, Elena-Raluca Nicoli, Joshi Stephen, David S. Yarnell, Heidi Dorward, William Owen, Suhag Parikh, Namik Yasar Ozbek, Baris Malbora, Carla Ciccone, Meral Gunay-Aygun, William A. Gahl, Wendy J. Introne, May Christine V. Malicdan

**Affiliations:** ^1^ Human Biochemical Genetics Section, Medical Genetics Branch, National Human Genome Research Institute, National Institutes of Health, Bethesda, MD, United States; ^2^ National Institutes of Health Undiagnosed Diseases Program, National Institutes of Health Common Fund, Office of the Director, National Institutes of Health, Bethesda, MD, United States; ^3^ Children’s Hospital of The King’s Daughters, Norfolk, VA, United States; ^4^ Department of Pediatrics, School of Medicine, Emory University, Atlanta, GA, United States; ^5^ Division of Pediatric Hematology and Oncology, University of Yeni Yuzyil, Gaziosmanpasa Hospital, Istanbul, Türkiye; ^6^ Department of Pediatric Hematology/Oncology, Ankara City Hospital, The University of Health Sciences, Ankara, Türkiye; ^7^ Office of the Clinical Director, National Human Genome Research Institute, National Institutes of Health, Bethesda, MD, United States

**Keywords:** LYST, rare disorders, monogenic diseases, oculocutaneous albinism, splicing abnormalities, personalized medicine, molecular diagnosis

## Abstract

**Introduction:** Chediak-Higashi syndrome (CHS) is rare autosomal recessive disorder caused by bi-allelic variants in the Lysosomal Trafficking Regulator (*LYST*) gene. Diagnosis is established by the detection of pathogenic variants in *LYST* in combination with clinical evidence of disease. Conventional molecular genetic testing of *LYST* by genomic DNA (gDNA) Sanger sequencing detects the majority of pathogenic variants, but some remain undetected for several individuals clinically diagnosed with CHS. In this study, cDNA Sanger sequencing was pursued as a complementary method to identify variant alleles that are undetected by gDNA Sanger sequencing and to increase molecular diagnostic yield.

**Methods:** Six unrelated individuals with CHS were clinically evaluated and included in this study. gDNA Sanger sequencing and cDNA Sanger sequencing were performed to identify pathogenic *LYST* variants.

**Results:** Ten novel *LYST* alleles were identified, including eight nonsense or frameshift variants and two in-frame deletions. Six of these were identified by conventional gDNA Sanger sequencing; cDNA Sanger sequencing was required to identify the remaining variant alleles.

**Conclusion:** By utilizing cDNA sequencing as a complementary technique to identify *LYST* variants, a complete molecular diagnosis was obtained for all six CHS patients. In this small CHS cohort, the molecular diagnostic yield was increased, and canonical splice site variants identified from gDNA Sanger sequencing were validated by cDNA sequencing. The identification of novel *LYST* alleles will aid in diagnosing patients and these molecular diagnoses will also lead to genetic counseling, access to services and treatments and clinical trials in the future.

## 1 Introduction

Chediak-Higashi syndrome (CHS, OMIM 214500) is a rare autosomal recessive disorder characterized by partial oculocutaneous albinism, bleeding diathesis, recurrent infections, an increased risk of developing the hyperinflammatory condition called hemophagocytic lymphohistiocytosis (HLH), and neurologic impairment (Béguez César, 1943; Chediak, 1952; Higashi, 1954; Introne et al., 1999; Toro et al., 2009; Shirazi et al., 2019; Sharma et al., 2020). The presence of giant intracytoplasmic granules in leukocytes on peripheral blood smear is pathognomonic for CHS and establishes a clinical diagnosis (Toro et al., 2009). The disorder exhibits high variable expressivity, ranging from early onset classical CHS to later onset atypical CHS; those with atypical CHS often have subtle clinical features and are likely underdiagnosed (Weisfeld-Adams et al., 2013; Introne et al., 2016). Current treatment includes chemotherapy and continuation therapy for the HLH, followed by allogenic hematopoietic stem cell transplantation (HSCT) to treat the hematological and immunological dysfunction (Eapen et al., 2007; Toro et al., 2009; Umeda et al., 2016), and levodopa for patients who develop parkinsonism (Hauser et al., 2000; Toro et al., 2009; Bhambhani et al., 2013).

CHS has significant clinical overlap with several other genetic disorders, emphasizing the importance of a molecular diagnosis. Other disorders that include gray or silvery hair as observed in CHS include Griscelli syndrome (MIM 214450, MIM 607624, MIM 609227), Elejalde neuroectodermal melanolysosomal syndrome (MIM 256710), and oculocerebral syndrome with hypopigmentation (MIM 257800) (Gironi et al., 2019). There are also other disorders that have phenotypic overlap with one clinical aspect of CHS such as familial HLH or oculocutaneous albinism. Additionally, there are other multisystemic disorders with phenotypic overlap with several clinical features of CHS, such as Vici syndrome (MIM 242840) characterized by hypopigmentation, combined immunodeficiency, cardiomyopathy, cataracts, corpus callosum agenesis, microcephaly, and failure to thrive (Dafsari et al., 1993), and Hermansky-Pudlak syndrome (MIM 203300, MIM 608233, MIM 614072, MIM 614073, MIM 614074, MIM 614075, MIM 614076, MIM 614077, MIM 614171, MIM 617050, and MIM 619172) characterized by oculocutaneous albinism, a bleeding diathesis, neutropenia, granulomatous colitis, and pulmonary fibrosis (Huizing et al., 1993). These disorders can be distinguished from CHS due to differences in phenotypic presentation, the absence of giant intracytoplasmic granules in leukocytes, and distinct molecular causes.

The Lysosomal Trafficking Regulator (*LYST*) gene is the only known molecular cause of CHS and bi-allelic pathogenic variants in *LYST* establish a molecular diagnosis (Barbosa et al., 1996; Nagle et al., 1996; Toro et al., 2009). *LYST* has 53 exons within a 13,466 bp transcript spanning approximately 222 kb of genomic DNA and encodes a 429 kDa putative lysosomal trafficking protein. The key challenges to establishing a molecular diagnosis for individuals with CHS include the large size of *LYST* that can influence interpretation of variant pathogenicity, and the paucity of common pathogenic variants. Conventional molecular genetic testing of *LYST* by Sanger sequencing of genomic DNA (gDNA) detects the majority of pathogenic variants, but for some individuals clinically diagnosed with CHS, pathogenic variants remain undetected, likely due to large-scale indels and deep intronic variants or synonymous exonic variants that lead to aberrant splicing. Uniparental disomy leading to the inheritance of two copies of a pathogenic *LYST* variant from one parent has also been reported as a molecular mechanism for CHS (Dufourcq-Lagelouse et al., 1999; Manoli et al., 2010; Boluda-Navarro et al., 2021).

Several strategies have been used to sequence *LYST*. These strategies include conventional gDNA Sanger sequencing (Toro et al., 2009), cDNA Sanger sequencing (Boluda-Navarro et al., 2021), the protein truncation test followed by cDNA sequencing to screen for nonsense and frameshift variants and to identify substitutions and small indels in *LYST* (Certain et al., 2000), as well as next-generation sequencing approaches such as exome sequencing and genome sequencing, particularly if other candidate loci are being considered as part of the differential diagnosis (Jin et al., 2017).

In this study, we aimed to provide a complete molecular diagnosis for six individuals with CHS by utilizing both conventional gDNA Sanger sequencing and cDNA Sanger sequencing. cDNA Sanger sequencing was chosen as a complementary method of variant detection since patient *LYST*-expressing dermal fibroblasts were available and because this method has been shown to effectively detect variant alleles that may not be readily detectable by conventional gDNA Sanger sequencing (Certain et al., 2000; Boluda-Navarro et al., 2021). We hypothesize that this strategy will aid in the identification of variants in this technically challenging gene to better molecularly diagnose individuals with CHS in the future.

## 2 Materials and methods

### 2.1 Patients and clinical evaluation

Six patients clinically diagnosed with classical or atypical CHS were included in this study. These patients were enrolled over the course of 10–20 years in the Investigation of Chediak-Higashi Syndrome and Related Disorders protocol 00-HG-0153 (NCT00005917, clinicaltrials.gov) or the Diagnosis and Treatment of Patients with Inborn Errors of Metabolism or Other Genetic Disorders protocol 76-HG-0238 (NCT00369421, clinicaltrials.gov) approved by the National Human Genome Research Institute’s Institutional Review Board. Written informed consent was obtained prior to the study. Clinical evaluation included collecting the medical and family history, physical examination, complete blood count (CBC) blood test. Peripheral blood smear analysis and microscopic hair examination were used to assess the presence of giant inclusions in leukocytes and pigment clumping in hair shafts, pathognomonic features of CHS.

### 2.2 Primary cell culture

Primary dermal fibroblasts or melanocytes from CHS patients were cultured from forearm skin biopsies using standard protocols. Further details are presented in the Supplementary Data. Unaffected control primary fibroblasts and melanocytes were obtained from Coriell Institute for Medical Research and American Type Culture Collection. Details of the unaffected control primary cells used in the study are noted in Supplementary Table S1.

### 2.3 Immunofluorescence microscopy analysis

Indirect immunofluorescence detection of the late endosomal and lysosomal membrane marker LAMP3/CD63 was performed in cultured fibroblasts and melanocytes as previously described (Yarnell et al., 2020). Further details are presented in the Supplementary Data.

### 2.4 Gene expression analysis

Total RNA was extracted from cultured primary dermal fibroblasts or melanocytes using the RNeasy Mini Kit (Qiagen). Genomic DNA was removed by on-column DNase I digestion (Qiagen) during total RNA extraction. Reverse transcription was performed using the Omniscript Reverse Transcription Kit (Qiagen) and Oligo (dT)_23_ primers (O4387, Sigma-Aldrich). Quantitative PCR was performed using TaqMan gene expression assays according to the manufacturer’s specifications. Further details are presented in the Supplementary Data.

### 2.5 Genomic DNA Sanger sequencing

Genomic DNA (gDNA) was extracted from peripheral blood or cultured primary dermal fibroblasts using standard procedures. gDNA from cultured primary fibroblasts was used if the gDNA from peripheral blood was no longer available. Regions of the *LYST* (NM_000081.3) coding sequence (exons 3–53), including intron-exon boundaries, were amplified by PCR using the primers listed in Supplementary Table S2. The size and specificity of the PCR products were confirmed by agarose gel electrophoresis. Sanger sequencing was performed using the BigDye Terminator v3.1 Cycle Sequencing Kit (Applied Biosystems) and separated on the 3130xl Genetic Analyzer (Applied Biosystems). Chromatograms were analyzed using Sequencher v5.0 software (Gene Codes Corporation).

### 2.6 cDNA Sanger sequencing

Total RNA was extracted from cultured primary dermal fibroblasts or melanocytes using the RNeasy Mini Kit (Qiagen). cDNA was synthesized by reverse transcription with the High-Capacity RNA-to-cDNA Kit (Applied Biosystems). Regions of the *LYST* coding sequence (exons 3–53) were amplified by PCR using the primers listed in Supplementary Table S3. PCR amplicons were generated and confirmed by agarose gel electrophoresis prior to Sanger sequencing as described above.

### 2.7 Molecular cloning

PCR products were cloned into the pCR4-TOPO TA vector using the TOPO TA Cloning Kit (Invitrogen). Single clones were Sanger sequenced to individually analyze the cDNA alleles for which chromatograms obtained from the initial cDNA Sanger sequencing were difficult to interpret.

### 2.8 ACMG-AMP classification

Variant pathogenicity classification was performed according to the standards and guidelines presented by the ACMG-AMP and the ACGS (Richards et al., 2015; Ellard et al., 2020). Further details are presented in the Supplementary Data.

### 2.9 Statistical analysis

Statistics were performed using GraphPad Prism 8 version 8.4.3 (GraphPad Software, San Diego, CA). A two-tailed Mann-Whitney test was performed for experiments comparing two sets of data. A *p*-value of less than 0.05 was considered statistically significant.

## 3 Results

### 3.1 Clinical findings

**FIGURE 1:**
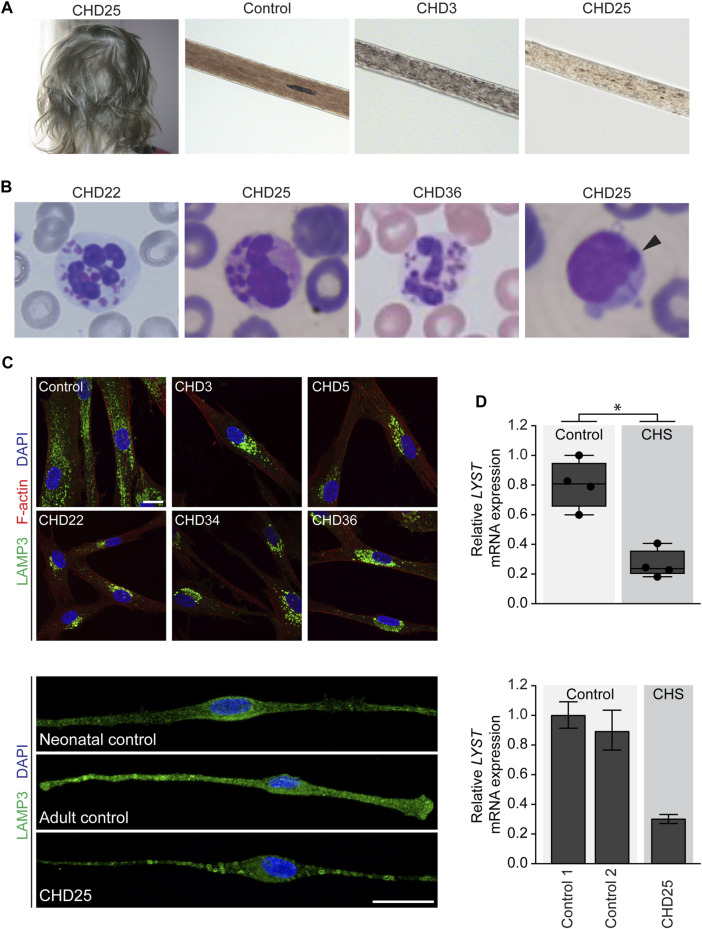
Clinical images of individuals with Chediak-Higashi syndrome (CHS) and cellular phenotype and *LYST* gene expression in primary cells from individuals with CHS **(A)** Partial oculocutaneous albinism often manifests as silvery hair and atypical pigment clumping of the hair shaft observed on light microscopy in individuals with CHS. **(B)** Peripheral blood smear demonstrating pathognomonic giant inclusions in polymorphonuclear leukocytes in CHD22, CHD25, and CHD36 and a single giant inclusion in a lymphocyte in CHD25 (arrowhead) **(C)** Primary fibroblasts (upper panel) and melanocytes (lower panel) were stained with LAMP3/CD63 antibodies to visualize lysosomal membranes. Primary fibroblasts were stained with phalloidin to visualize cell boundaries marked by F-actin. Lysosomes in unaffected control fibroblasts are distributed throughout the cell, while fibroblasts from individuals with CHS show enlarged lysosomes restricted to the perinuclear area. In melanocytes from CHD25, enlarged lysosomes were distributed throughout the cell compared to unaffected control melanocytes. Scale bar = 20 microns **(D)** Relative *LYST* mRNA expression in primary fibroblasts (upper panel) or melanocytes (lower panel). For the fibroblast expression data, the data are presented as a box and whisker plot relative to the highest *LYST*-expressing unaffected control with all individual mean data points represented. Group relative *LYST* expression differences were tested using a two-tailed Mann-Whitney test. For the melanocyte expression data, the data are presented as the mean of three technical replicates relative to the highest *LYST*-expressing unaffected control and error bars represent one standard deviation. Expression of *HPRT1*, *POLR2A*, and *TBP* were used as internal controls to normalize gene expression. Abbreviations: *, *p* < 0.05.

CHD3 presented with silvery hair, pale skin, and ocular albinism as well as a history of recurrent respiratory and skin infections and nose bleeds. He was clinically diagnosed with classical CHS at the age of 7 years. Abnormal pigment clumping was observed in his hair shafts (Figure 1A). He received a HSCT at age 9 years following HLH. Progressive neurological dysfunction with dementia was noted at 21 years of age.

CHD5 has atypical CHS and was previously reported (Introne et al., 2016). She was diagnosed at the age of 19 years and presented with relatively mild disease manifesting as ocular albinism, gingival bleeding, and cutaneous methicillin-resistant *Staphylococcus aureus* (MRSA). She also had learning difficulties and a mood disorder. Pathognomonic giant inclusions were observed in her leukocytes and abnormal pigment clumping in her hair shafts (Introne et al., 2016).

CHD22 was hospitalized at 12 days of life due to omphalitis. She had recurrent sinopulmonary infections and otitis media, severely diminished platelet dense bodies, and patchy areas of hyper- and hypopigmentation of the skin. The presence of the pathognomonic giant cytoplasmic inclusions was confirmed by peripheral blood smear (Figure 1B), and she was clinically diagnosed with classical CHS at 1 year of age. She underwent HSCT at 1 year 8 months of age.

CHD25 was a 3.5-year-old female from Northern Turkey with a history of frequent bronchopulmonary infections who was admitted to the hospital due to persistent fever. Her mother and father were third degree relatives. She had one brother who was born at the 8th gestational month and died of an unknown cause in infancy. She also presented with silvery, hypopigmented hair, pale skin, and ocular albinism (Figure 1A). Peripheral blood smear confirmed giant cytoplasmic granules within the granulocytes, and lymphocytes showed a single giant inclusion (Figure 1B). She was clinically diagnosed with classical CHS. At presentation, she had evidence of recent Epstein-Barr Virus (EBV) infection and pancytopenia suggestive of HLH. Etoposide and methylprednisolone were started to treat her HLH. She had two posterior reversible leukoencephalopathy syndrome (PRES) attacks during the treatment and died due to respiratory failure during the second PRES attack, which involved the respiratory center of the pons.

CHD34 was diagnosed with pneumonia at birth and peripheral blood screening revealed giant granules pathognomonic for CHS. He was protected from infections until HSCT at 8 months of age and had no further infections or abnormal bleeding prior to transplant. He had a history of ocular albinism and patchy hyper- and hypopigmentation of the skin.

CHD36 had persistent thrush in the neonatal period that was unresponsive to nystatin and treated with two courses of fluconazole. She also had delayed separation of the umbilical cord and two episodes of respiratory infections. Upon referral to a dermatologist for an area of alopecia, she was noted to have silvery hair and patchy hypopigmentation of the skin, suggestive of CHS. Giant granules within the leukocytes were confirmed by blood smear (Figure 1B), and she was clinically diagnosed with classical CHS. She also had iris transillumination and platelet dense granule deficiency. She received HSCT at 10 months of age.

### 3.2 Cellular findings

To further support the clinical diagnosis of these six individuals, we pursued additional studies including assessment of lysosome morphology and intracellular localization by indirect immunofluorescence and relative *LYST* gene expression by quantitative PCR in cultured primary dermal fibroblasts or melanocytes. Indirect immunofluorescence staining of the late endosomal and lysosomal membrane marker LAMP3/CD63 in CHS fibroblasts and melanocytes revealed enlarged lysosomes (Figure 1C). Perinuclear localization of these lysosomes was apparent in CHS fibroblasts, while the enlarged lysosomes were located throughout CHD25 melanocytes. Quantitative PCR demonstrated reduced relative *LYST* mRNA expression in CHS fibroblasts and melanocytes compared to unaffected controls (Figure 1D, *p*-value = 0.0286 for fibroblast data). These cellular findings substantiate prior cellular findings reported in human CHS and in the *beige* mouse model (Perou et al., 1997; Westbroek et al., 2007) and support the clinical diagnosis of CHS in these six individuals.

### 3.3 Molecular genetic findings

**FIGURE 2:**
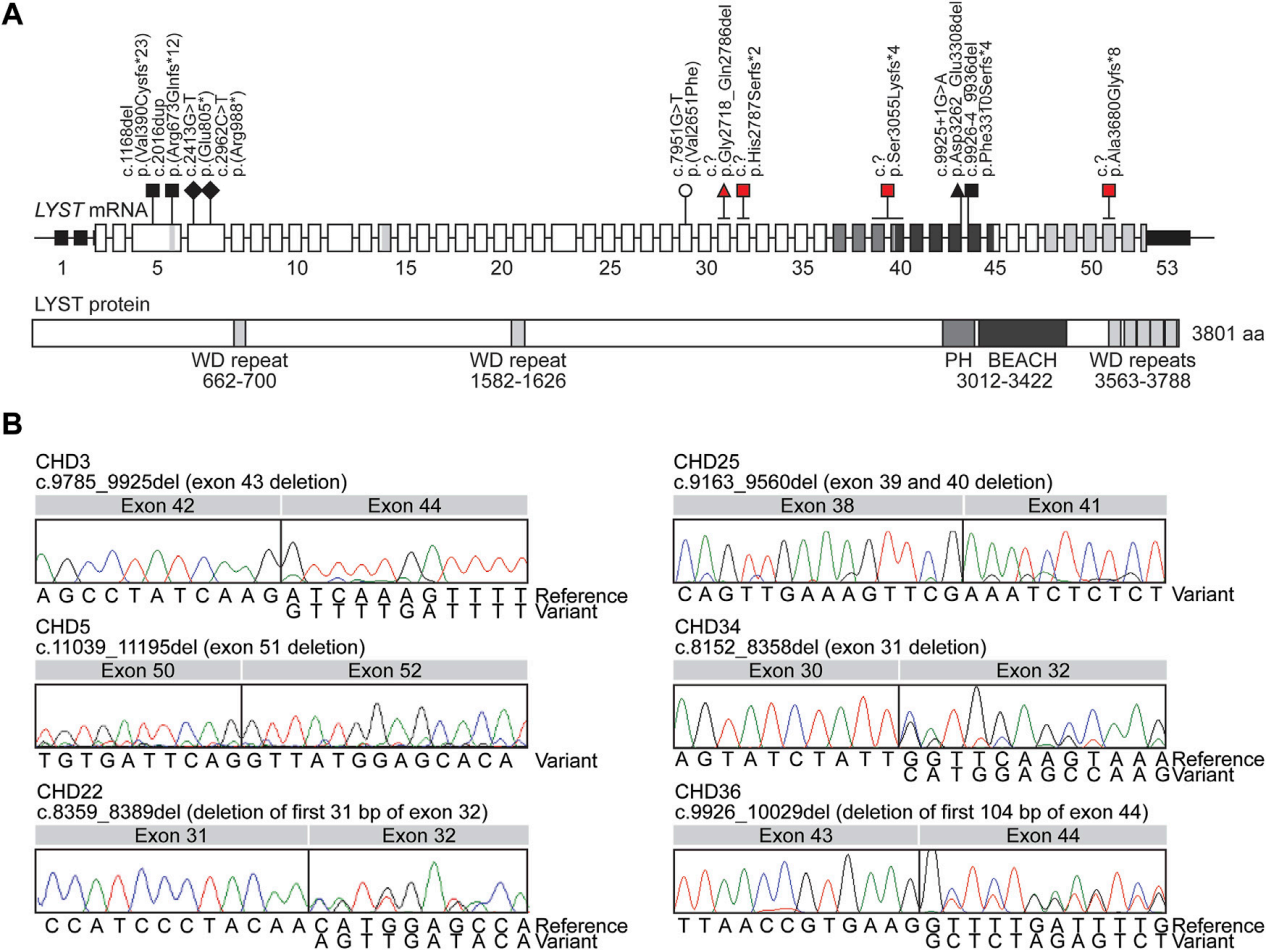
*LYST* variant alleles in individuals with Chediak-Higashi syndrome (CHS) identified by gDNA and cDNA Sanger sequencing **(A)** Schematics of the *LYST* mRNA (NM_000081.3) and LYST protein (NP_000072.2) denoting the relative locations of the *LYST* variants identified by gDNA and cDNA Sanger sequencing. Previously reported variants (white), novel variants identified by gDNA Sanger sequencing (black), and novel variants identified by cDNA Sanger sequencing (red) are represented in the schematic and include frameshift (square),nonsense (diamond), missense (circle), and in-frame (triangle) variants. Functional domains of LYST include the WD repeats (light grey), the PH domain (medium grey), and the BEACH domain (dark grey), and the corresponding regions are color-coded in the *LYST* mRNA schematic. The *LYST* mRNA schematic is not drawn to scale, while the LYST protein schematic is drawn to scale **(B)** Chromatograms generated through cDNA Sanger sequencing of *LYST*, including the c.9785_9925del (exon 43 deletion) variant allele identified in CHD3, the c.11039_11195del (exon 51 deletion) variant allele identified in CHD5, the c.8359_8389del (partial exon 32 deletion) variant allele identified in CHD22, the homozygous c.9163_9560del (exon 39 and 40 deletion) variant allele identified in CHD25, the c.8152_8358del (exon 31 deletion) variant allele identified in CHD34, and the c.9926_10029del (partial exon 44 deletion) variant allele identified in CHD36. RT-PCR products were cloned to obtain sequence data for the c.11039_11195del variant allele. Abbreviations: BEACH domain, beige and Chediak-Higashi syndrome domain; *LYST*, lysosomal trafficking regulator; PH domain, pleckstrin homology domain.

To identify *LYST* variants in these six individuals with clinically confirmed CHS, conventional gDNA Sanger sequencing of the exonic regions of *LYST* was first performed. One or two variants were identified for CHD3, CHD5, CHD22, CHD34, and CHD36 (Figure 2A; Table 1). CHD3 was found to have a novel c.2413G>T variant predicted to lead to a p.(Glu805*) nonsense variant and a novel c.9925+1G>A variant predicted to affect the canonical splice donor site of intron 43. CHD5 was previously found to have a heterozygous c.7951G>T, p.(Val2651Phe) missense variant (Introne et al., 2016). CHD22 was found to have a novel c.1168del variant predicted to lead to a p.(Val390Cysfs*23) frameshift variant. CHD34 had a novel c.2016dup variant predicted to lead to a p.(Arg673Glnfs*12) frameshift variant. CHD36 had a novel c.2962C>T variant predicted to lead to a p.(Arg988*) nonsense variant and a novel c.9926-4_9936del variant predicted to affect the canonical splice acceptor site of intron 43. While no variants were identified for CHD25, PCR amplicons overlapping exons 39 and 40 failed to amplify, suggesting the possibility of a homozygous deletion in this region.

**TABLE 1:** Summary of variants identified in *LYST* (NM_000081.3, reference genome GRCh37/hg19) by gDNA and cDNA Sanger sequencing in six individuals with CHS.

Patient ID	Coding sequence change from gDNA Sanger sequencing	Coding sequence change from cDNA Sanger sequencing	Amino acid change	ACMG-AMP classification	References
CHD3^a^	c.2413G>T	NA	p.(Glu805*)	Pathogenic (PVS1, PM2, PP4)	This study
	c.9925+1G>A	c.9785_9925del (exon 43 deletion)	p.Asp3262_Glu3308del	Likely pathogenic (PVS1_Strong^b^, PM2, PP4)	This study
CHD5	c.7951G>T	NA	p.(Val2651Phe)	Likely pathogenic (PM2, PM3, PP3, PP4)	Gil-Krzewska et al. (2016), Introne et al. (2017)
	c.?^c^	c.11039_11195del (exon 51 deletion)	p.Ala3680Glyfs*8	Uncertain significance (PVS1, PP4)	This study
CHD22	c.1168del	NA	p.(Val390Cysfs*23)	Pathogenic (PVS1, PM2, PM3, PP4)	This study
	c.?^d^	c.8359_8389del (partial exon 32 deletion)	p.His2787Serfs*2	Pathogenic (PVS1, PM3, PP4)	This study
CHD25	c.?^e^	c.9163_9560del (exon 39 and 40 deletion)	p.Ser3055Lysfs*4	Pathogenic (PVS1, PM3, PP4)	This study
CHD34^a^	c.2016dup	NA	p.(Arg673Glnfs*12)	Pathogenic (PVS1, PM2, PP4)	This study
	c.?^f^	c.8152_8358del (exon 31 deletion)	p.Gly2718_Gln2786del	Uncertain significance (PVS1_Moderate, PP4)	This study
CHD36	c.2962C>T	NA	p.(Arg988*)	Pathogenic (PVS1, PM2, PM3, PP4)	This study
	c.9926-4_9936del	c.9926_10029del (partial exon 44 deletion^g^)	p.Phe3310Serfs*4	Pathogenic (PVS1, PM2, PM3, PP4)	This study

Abbreviation: NA, not applicable.

^a^ Parental DNAs, were not available for these CHS patients and, therefore, it is unknown whether these variants are in *trans* and the PM3 evidence cannot be applied as part of the ACMG-AMP classification.

^b^ This in-frame deletion overlaps the BEACH, domain (amino acids 3132–3422), a region critical to LYST, function.

^c^ DNA, Sanger sequencing identified deletion of exon 51 likely due to aberrant splicing resulting in exon skipping, however the causative gDNA, change could not be determined.

^d^ cDNA, Sanger sequencing identified deletion of the first 31 bp of exon 32 likely due to aberrant splicing resulting in partial exon skipping, however the causative gDNA, change could not be determined.

^e^ cDNA, Sanger sequencing identified deletion of exons 39 and 40, however the gDNA, change could not be determined. Exons 39 and 40 could not be amplified from the gDNA, suggesting a homozygous deletion in this region.

^f^ cDNA, Sanger sequencing identified deletion of exon 31 likely due to aberrant splicing resulting in exon skipping, however the causative gDNA, change could not be determined.

^g^ cDNA, Sanger sequencing identified deletion of the first 104 bp of exon 44 due to aberrant splicing resulting in partial exon skipping.

To further identify *LYST* alleles, cDNA Sanger sequencing of the coding regions of *LYST* was performed. Four additional variant alleles were identified, and cDNA-level consequences were revealed for the two splice site variants (Figure 2; Table 1). CHD5 was found to have a novel c.11039_11195del variant allele (deletion of exon 51) that is predicted to lead to a p.Ala3680Glyfs*8 frameshift variant; CHD22 had a novel c.8359_8389del variant allele (deletion of the first 31 bp of exon 32) predicted to lead to a p.His2787Serfs*2 frameshift variant. CHD25 had a novel homozygous c.9163_9560del variant allele (deletion of exons 39 and 40) that is predicted to lead to a p.Ser3055Lysfs*4 frameshift variant. Finally, CHD34 was found to have a novel c.8152_8358del variant allele (deletion of exon 31) that is predicted to lead to a p.Gly2718_Gln2786 in-frame deletion. Variants at the level of gDNA could not be determined. For the canonical splice site variants in CHD3 and CHD36, the c.9925+1G>A variant led to c.9785_9925del (deletion of exon 43), confirming an in-frame variant (p.Asp3262_Glu3308del), and the c.9926-4_9936del variant led to c.9926_10029del (partial deletion of exon 44), confirming a frameshift variant (p.Phe3310Serfs*4). These findings establish a molecular diagnosis of CHS for all these individuals.

## 4 Discussion

In this study, we performed detailed clinical phenotyping and gDNA Sanger sequencing complemented by cDNA Sanger sequencing to establish a complete clinical and molecular diagnosis for six individuals with CHS. We report ten novel *LYST* alleles that may aid the molecular diagnosis of patients in the future; four of these variant alleles were undetectable by conventional gDNA Sanger sequencing and subsequently identified by cDNA Sanger sequencing.

Pathogenic variants have been identified throughout the *LYST* gene with no hotspots or hypermutated regions, and the majority of reported pathogenic variants in *LYST* have been nonsense or frameshift variants (personal communication) (Toro et al., 2009; Sanchez-Guiu et al., 2014). Eight of the ten novel variants reported in this study are predicted to lead to a nonsense or frameshift variant, while the remaining two novel variants are predicted to lead to an in-frame deletion (Figure 2; Table 1). Individuals with classical CHS often have bi-allelic nonsense or frameshift variants, while individuals with atypical CHS often have at least one missense variant, although exceptions have been reported (personal communication) (Toro et al., 2009; Sanchez-Guiu et al., 2014). Although CHD34 presented with classical CHS, one of his variants leads to an in-frame deletion of exon 31 (p.Asp3262_Glu3308del) and, while there are no known functional domains in this region of LYST, this region may represent a critical domain important for LYST function. Interestingly, CHD3 also presented with classical CHS and, in addition to a nonsense variant, CHD3 had an in-frame deletion within the BEACH domain (p.Gly2718_Gln2786del) that is critical for LYST function.

cDNA Sanger sequencing in combination with conventional gDNA Sanger sequencing offers several advantages. First, cDNA Sanger sequencing can detect variant alleles that are not readily detectable by conventional gDNA Sanger sequencing. While establishing a clinical diagnosis is valuable, the confirmation of a molecular diagnosis is critical for optimal patient care, genetic counseling for the individual and their family members, and eligibility in future clinical trials. Second, cDNA Sanger sequencing is relatively easy to incorporate into the molecular genetic testing workflow, requiring only routine cell culture and total RNA extraction techniques. An important consideration is patient sample archiving of *LYST*-expressing cell types, including dermal fibroblasts, melanocytes, NK cells, and lymphoblastoid cell lines, for the preparation of total RNA. Archiving cells from each parent and all available family members would also be useful for variant phasing and segregation studies.

A key limitation of this study was that we were unable to identify the cause of the full or partial exon skipping at the gDNA level for some alleles. While efforts were made to sequence nearby intronic regions and identify exonic synonymous variants that might lead to the aberrant splicing, proving the consequence of such variants requires further functional validation. With the decreasing costs of genome sequencing and targeted next-generation sequencing, these are becoming viable options for the identification of deep intronic variants leading to splicing changes in *LYST*. Furthermore, only cDNA from each of the six patients was analyzed in this study due to sample availability. While we were unable to assess cDNA from cells derived from family members due to sample availability, cDNA Sanger sequencing could be performed to segregate *LYST* alleles in a family even if the variant at the level of gDNA is unknown.

In this study, the integration of thorough clinical phenotyping and conventional gDNA Sanger sequencing in combination with cDNA Sanger sequencing increased the mutational spectrum of CHS. Based on our experience from this study, we provide the following recommendations for molecular genetic testing of the *LYST* gene in CHS. Because of the increasing access to next-generation sequencing and its rapid turnaround time, we recommend the utilization of clinical genome sequencing to include copy number variant analysis for patients in which CHS is suspected. When available, RNA sequencing using lymphoblastoid cell lines and melanocytes could help delineate the resulting splice site abnormalities from intronic variants. For standard clinical practice using exome sequencing and when clinical genome sequencing is not available, targeted gDNA Sanger sequencing and copy number detection is recommended for patients highly suspected to have clinical CHS, followed by RNA sequencing (as above) or cDNA Sanger sequencing if only one or no variants are detected by gDNA Sanger sequencing. It will also be interesting to pursue efforts on long-read sequencing technologies to identify deletions in these large genes and to phase variants in the absence of parental samples in the future. Overall, the identification of disease-causing variants will aid in the future diagnosis and care of individuals with CHS.

## Data Availability

The original contributions presented in the study are included in the article/Supplementary Material, further inquiries can be directed to the corresponding author.

## Abbreviations

CBC, complete blood count; CHS, Chediak-Higashi syndrome; EBV, Epstein-Barr virus; HLH, hemophagocytic lymphohistiocytosis; HSCT, hematopoietic stem cell transplantation; LYST, Lysosomal Trafficking Regulator; MRSA, methicillin-resistant *Staphylococcus aureus*; PRES, posterior reversible leukoencephalopathy syndrome.
