# Supplementary material for: cDNA sequencing increases the molecular diagnostic yield in Chediak-Higashi syndrome
Source: Front Genet. 2023 Mar 8;14:1072784. doi: 10.3389/fgene.2023.1072784 (PMC10031035; doi:10.3389/fgene.2023.1072784)
Supplement: Supplementary file 1 [file DataSheet1.docx]

**SUPPLEMENTARY DATA**

**cDNA sequencing increases the molecular diagnostic yield in Chediak-Higashi syndrome**

Chulaluk Kuptanon, Marie Morimoto, Elena-Raluca Nicoli, Joshi Stephen, David S. Yarnell, Heidi Dorward, William Owen, Suhag Parikh, Namik Yasar Ozbek, Baris Malbora, Carla Ciccone, Meral Gunay-Aygun, William A. Gahl, Wendy J. Introne, May Christine V. Malicdan

**SUPPLEMENTARY METHODS**

**2.2. Primary cell culture**

Primary dermal fibroblasts or melanocytes from CHS patients were cultured from forearm skin biopsies using standard protocols. Fibroblasts were cultured in high glucose DMEM with 10% fetal bovine serum and 1× antibiotic-antimycotic (Gibco) at 37°C with 5% CO_2_. Melanocytes were cultured in DMEM/F-12, GlutaMAX supplement (10565018, Gibco) with 5% FBS, 50 nM 12-O-tetradecanoylphorbol 13-acetate (TPA) (P8139, Sigma-Aldrich), 100 pM cholera toxin (C8052, Sigma-Aldrich), 5 ng/ml basic fibroblast growth factor (F0291, Sigma-Aldrich), 1 nM endothelin 1 (E7764, Sigma-Aldrich), 1 mM L-ascorbic acid (A4403, Sigma-Aldrich), 1× MEM non-essential amino acids (11140050, Gibco), 1× insulin-transferrin-selenium (41400045, Gibco), and 1× antibiotic-antimycotic at 37°C with 5% CO_2_. Unaffected control primary fibroblasts and melanocytes were obtained from Coriell Institute for Medical Research and American Type Culture Collection.

**2.3. Immunofluorescence microscopy analysis**

Indirect immunofluorescence detection of the late endosomal and lysosomal membrane marker LAMP3/CD63 was performed in cultured fibroblasts and melanocytes as previously described (Yarnell et al., 2020). Mouse monoclonal LAMP3/CD63 antibody (H5C6, Developmental Studies Hybridoma Bank) was used as a primary antibody and an Alexa Fluor 488-conjugated donkey anti-mouse IgG antibody was used as a secondary antibody (Invitrogen). Alexa Fluor 555 Phalloidin (Invitrogen) was used to visualize the whole cell for fibroblast staining. Cells were mounted in VectaShield Antifade Mounting Medium with DAPI (Vector Laboratories). Images were acquired using an LSM 880 laser scanning confocal microscope with a 63× oil immersion lens using the Zen Black software (Carl Zeiss Microscopy).

**2.4. Gene expression analysis**

Total RNA was extracted from cultured primary dermal fibroblasts or melanocytes using the RNeasy Mini Kit (Qiagen). Genomic DNA was removed by on-column DNase I digestion (Qiagen) during total RNA extraction. Reverse transcription was performed using the Omniscript Reverse Transcription Kit (Qiagen) and Oligo(dT)_23_ primers (O4387, Sigma-Aldrich).

Quantitative PCR was performed using a TaqMan gene expression assay that detects both isoforms of *LYST* (Hs00915889_m1, Applied Biosystems), the TaqMan Gene Expression Master Mix (Applied Biosystems), and the 7500 Fast Real-Time PCR System (Applied Biosystems). Expression of the following genes was used as the internal control to normalize gene expression: *HPRT1* (Hs02800695_m1), *POLR2A* (Hs00172187_m1), and *TBP* (Hs00427620_m1) (Applied Biosystems). Each TaqMan assay was performed in triplicate and the following conditions were used for amplification: 1 cycle of 50°C for 2 min for uracil-N-glycosylase incubation, followed by 1 cycle of 95°C for 10 min for DNA polymerase activation, followed by 40 cycles of 95°C for 15 s and 60°C for 1 min for PCR amplification. The relative quantification of gene expression was calculated using the delta-delta C_t_ method with the 7500 Software version 2.3 (Applied Biosystems) (Livak and Schmittgen, 2001). For the melanocyte samples, bovine serum albumin was added to a final concentration of 0.8 mg/ml to the reverse transcription reaction and to the quantitative PCR to reverse the inhibition of DNA polymerase by melanin that co-purifies with nucleic acids (Giambernardi et al., 1998; Eckhart et al., 2000).

**2.8. ACMG-AMP classification**

The strength of the PVS1 evidence for null variants was adjusted according to published recommendations (Abou Tayoun et al., 2018). The PM2 criteria was applied if the variant frequency was <0.1% in gnomAD. The PM3 criteria was applied if the variant was detected in *trans* with a null allele (nonsense, frameshift, canonical 1 or 2 splice sites, initiation codon, single or multiexon deletion). MutationTaster, PolyPhen-2 (HVarPred), SIFT, and CADD phred scores were used to assess the potential impact of *LYST* missense variants. If at least 3 of the 4 scores were supportive, then the PP3 evidence was included. The presence of enlarged intracytoplasmic granules on peripheral blood smear was required to include the PP4 evidence for phenotype specificity. ClinVar was not considered a reputable source for the PP5 and BP6 evidence according to published recommendations (Biesecker et al., 2018).

**SUPPLEMENTARY TABLES**

**Supplementary Table 1.** Characteristics of the unaffected controls used in this study.

| **ID** | **Lot number** | **Company** | **Cell type** | **Sex** | **Age at sampling** |
| --- | --- | --- | --- | --- | --- |
| GM00942^1^ | NA | CIMR | Fibroblast | Female | 5 years |
| GM01651^2^ | NA | CIMR | Fibroblast | Female | 13 years |
| GM09503^2^ | NA | CIMR | Fibroblast | Male | 10 years |
| PCS-201-010^2^ | 60235894 | ATCC | Fibroblast | Male | Neonate |
| PCS-201-012^2^ | 8221500 | ATCC | Fibroblast | Female | 52 years |
| PCS-200-012^1,2^ | 58349697 | ATCC | Melanocyte | Male | Neonate |
| PCS-200-013^1,2^ | 80626173 | ATCC | Melanocyte | Female | 47 years |

^1^Unaffected control used in the immunofluorescence microscopy analysis presented in Figure 1C.

^2^Unaffected control used in the gene expression analysis presented in Figure 1D.

Abbreviations: ATCC, American Type Culture Collection; CIMR, Coriell Institute for Medical Research; NA, not applicable.

**Supplementary Table 2.** List of oligonucleotide primers for *LYST* gDNA Sanger sequencing.

| **Primer Name** | **Primer Sequence (5' to 3')** |
| --- | --- |
| LYST X3F | TTCCAGTAATTTTAAGCCAATCTTC |
| LYST X3R | TCTCAAGGAGGCTTCAGAAA |
| LYST X4F | GCTTTTCAAATGCAGACTTTCA |
| LYST X4R | CAGAATCAAACATTCAGGCAGA |
| LYST X5.1F | GAGGGCTGTCACCTTAAAATTG |
| LYST X5.1R | AGGCAGCTGGCTCACTTAAA |
| LYST X5.2F | CAAGACCACCCCAAAGCTAA |
| LYST X5.2R | AAGGGTCAGGCTGCTTTTCT |
| LYST X5.3F | TCTTTCGAACAGTGTTGCATCT |
| LYST X5.3R | CCGAAACCAGAAGACCTGAG |
| LYST X5.4F | TCAGAGCAACTTCATCATTCG |
| LYST X5.4R | GCAGGATCCCTTGAAATCTG |
| LYST X5.5F | TTGGATCAGTTAGGAGGAGCA |
| LYST X5.5R | CACTTGAAAGCATCCAATGAAA |
| LYST X6.1FA | TGGTTAGTATATCCCACATGCAA |
| LYST X6.1RA | GACCAAATGTCTGCTGCTTG |
| LYST X6.2F | CGGCCAATGACTCAGAAGAT |
| LYST X6.2R | CATAAGAGTTGGACTAAGGACAAGG |
| LYST X7F | CAACTGCAAATAAATGGATTAGCTT |
| LYST X7R | CGTCCTAGTGTCATCCCACA |
| LYST X8F | GAAACTGTCCTTAACATGACTTTTGA |
| LYST X8R | CACACTTGTTAAACTGTAAGGCAAT |
| LYST X9F | GCATCCTCTGAATTACTATTTTTGC |
| LYST X9R | GGAGATGTTATTGGGTGATGAG |
| LYST X10F | CATAGAATTTATTATGACATTGCAGTT |
| LYST X10R | ACCTGGCCAGAAGCCATTAT |
| LYST X11F | TGGTGAAATTGCTCATTTATTCC |
| LYST X11R | TGTCACATCAAAAGAAAGAGCTTA |
| LYST X12F | GGAATGCTGATATGTGTGGGTA |
| LYST X12R | TGTGTACAAATCAGATAATCCTGTCA |
| LYST X13F | TGAACAAATTTGCTTTGTATTCTTC |
| LYST X13R | TGAGACTTTTTGTTTTGCTATAATGAA |
| LYST X14F | AAACACTGAGGTCAGAATTTTCAT |
| LYST X14R | AACATTGTAACTGAGATTGAGATGC |
| LYST X15F | AAAAAGAATTCCCCATTGTTAAA |
| LYST X15R | TGGAAATGTTTCAAAGGAAAA |
| LYST X16F | TCCTGGTTTCTTGCTTTTTATGA |
| LYST X16R | AAGAAAATGATAAGGAACCACATT |
| LYST X17F | AAGGATCAGTTGAGTGTGTTTTCA |
| LYST X17R | AGCTTTTTCGTGTGGTCCAA |
| LYST X18F | TGGATTGATGTTACAGAATGGAA |
| LYST X18R | TTTCCTCCATTTTCTTCAGGA |
| LYST X19F | TTTCCTTTGAATTTAATGATTGC |
| LYST X19R | GCACTTAATGCCACTGAACTGA |
| LYST X20F | GCGTTGTGTGTCTTCCAAAA |
| LYST X20R | CCATTGAAAGTGCCACAAAA |
| LYST X21F | TTTTGCCTAAATGAAAAATCTTAGTT |
| LYST X21R | GGCCATTCAAATGTCAAACA |
| LYST X22F | CAAAATTTGAATTTAATGATCTGCTT |
| LYST X22R | TCTCTTGTTAGATTGAATGAGGTTG |
| LYST X23F | TGCAAAAGAGAGGTGGTTCC |
| LYST X23R | GAATAGTAAAATAAAGGTGGGAGGA |
| LYST X24F | GCATTATGAACATTTGTTTCACC |
| LYST X24R | CATTATTCTAAAATATAAAAGAGAGATTTAGG |
| LYST X25F | TCATTTCTGCTTTGCTGTGG |
| LYST X25RA | CCCAATTATAGTGGAGGGAAGA |
| LYST X26F | GCAAGATGACCCATAGTTGCT |
| LYST X26R | GAGGTACACAGAAAGGCTTCTTACA |
| LYST X27F | TGAATGAGTGCAAGCTGGTC |
| LYST X27R | ACAACTCCCTCCCCAAATTC |
| LYST X28F | TTGTCATGATACATAAAATCAGTTCAA |
| LYST X28R | TGTGAATGGCATAAGAACATAGG |
| LYST X29F | AAGGTTGTTTTCATGGTCTGG |
| LYST X29R | GAAAACCAACCAGTTTCCTCTT |
| LYST X30F | TTTATAATGGCAAGATAGCAGTGA |
| LYST X30R | CCTCTATTTCCTTTGTGTGAATGA |
| LYST X31F | CTGTGTTGAGGCTAGCATGG |
| LYST X31R | CAGACTTTGTATTTCACCAAACG |
| LYST X32F | TGCAACCAAATAAATCTAGCAA |
| LYST X32R | TCATCATCAATGATAGCCTGTTC |
| LYST X33-34F | TTTGTTGGGCTCTGTGACAA |
| LYST X33-34R | CCGGAATACAAATTCCGTTT |
| LYST X35-36F | TCAGTTGTGACTTACTTACTCAATCTC |
| LYST X35-36R | CCTTTCCTGTTTAGGATTTTTCTG |
| LYST X37F | GTCTGCTTTGCAGAAATCTTTT |
| LYST X37R | AAGGCAGGGTGAATATGTGC |
| LYST X38F | AAGGACTCCAGAGACCATGC |
| LYST X38R | TGAATTGATACATTTTTGGAATCTG |
| LYST X39F | GCCATCTGCCACAAAAAGAT |
| LYST X39R | TGGCCCATGAGCACTTAAAC |
| LYST X40F | TGTCATGGGTTCTTTGGAGA |
| LYST X40R | TTCTTCTATTTTGTGCATGTTTGAA |
| LYST X41F | GGTGGAGCATTTTGTTGACC |
| LYST X41R | CACAAGTCTGTAAAACAAAACACAA |
| LYST X42F | AATGGCCTCAAAGGGGTAGT |
| LYST X42R | TTGTTTCTGATGACCCAACA |
| LYST X43F | AATCTTTTTGAAGTGGCAAAAGT |
| LYST X43R | TTTGGAAAATTACAAAATAACCACA |
| LYST X44F | TGGTTGTAGCACACAAACAGC |
| LYST X44R | TAAAGGAATGGCCGAACAAC |
| LYST X45F | AAAGTGTTGTTTTTCCCTTTGG |
| LYST X45R | GCCAGGACCATGCTATTCAT |
| LYST X46F | GCCCTTACAGAGCTTATAATCCA |
| LYST X46R | GCTGAGTTTTTCCACCAAGG |
| LYST X47F | GATGGTCTTGCACCAAATGA |
| LYST X47R | AGGCTGAGGCAGGAGAATG |
| LYST X48F | TTTTAAAACATGATAATCTTTGATTGG |
| LYST X48R | AAGGCTTTCTTCCCCTCATT |
| LYST X49F | CCATTTGAGTCACTTTGGTTG |
| LYST X49R | CATGTGTTTCATGGTTAAAATGATTA |
| LYST X50F | TGAGAAGAAGAATTACAAAATAAGAGAATG |
| LYST X50R | CTGAGAGAATGGCTCGACCT |
| LYST X51F | CAAATGTGGGCTCTGAGACA |
| LYST X51R | TTTCTGAAACTCCTGTGTCCTT |
| LYST X52F | TGTTTGTTACATTTCCTTCCACA |
| LYST X52R | TCATGATGACTTCAATTGCACA |
| LYST X53F | TATGTCTCTCTGCGCCACTG |
| LYST X53R | TTGGATGGTTTGTCCAGTCA |

Abbreviations: F, forward; gDNA, genomic DNA; R, reverse.

**Supplementary Table 3.** List of oligonucleotide primers for *LYST* cDNA Sanger sequencing.

| **Primer Name** | **Location** | **Primer Sequence (5' to 3')** |
| --- | --- | --- |
| LYST X1-2F | Exons 1-2 | CCACAAACCAGGTGAAGC |
| LYST X1-2R | Exons 1-2 | GCAAGAAAGACACAGCTCTCC |
| LYST X2-5F | Exons 2-5 | TGCCACAAACCAGGTGAAGCT |
| LYST X2-5R | Exons 2-5 | CAGCTCTCCACCTCAGATTCAG |
| LYST X5aF | Exon 5a | TAACCTACCGCTCTCAGCAG |
| LYST X5aR | Exon 5a | AGCGACAACTTGGAGAGTCG |
| LYST X5bF | Exon 5b | TGGATCAGTTAGGAGGAGCA |
| LYST X5bR | Exon 5b | TTGCCTCAGGACGTGCTTC |
| LYST X5-6F | Exons 5-6 | CCTGTGCTCCAAAGAGGAGT |
| LYST X5-6R | Exons 5-6 | AGCCTTGTCTTGCCTTCTCC |
| LYST X5-10F | Exons 5-10 | AAGCCCTTCTGGCCATTTGT |
| LYST X5-10R | Exons 5-10 | GCTCATGCAACAGGGAACTG |
| LYST X6-7F | Exons 6-7 | AGAGTCTGACAGGGAGTCGG |
| LYST X6-7R | Exons 6-7 | GGGACCATCTTTCCCAGTCA |
| LYST X6-10F | Exons 6-10 | AAGCCCTTCTGGCCATTTGT |
| LYST X6-10R | Exons 6-10 | GCTCATGCAACAGGGAACTG |
| LYST X9-12F | Exons 9-12 | GCCAAACTTGATGTGCTTGC |
| LYST X9-12R | Exons 9-12 | ACAGAGAGCGACAGACCAGA |
| LYST X12-16F | Exons 12-16 | GGTTTCAGTGTTTCCCTGTGG |
| LYST X12-16R | Exons 12-16 | AGGTTGGTTCACAAGAGGCC |
| LYST X14-17F | Exons 14-17 | TCTGGACAGAGGAAGCCTGA |
| LYST X14-17R | Exons 14-17 | TGCCAGGGTTGTTGAACTCA |
| LYST X17-20F | Exons 17-20 | ACCCAACTCTCTCAAAGACCC |
| LYST X17-20R | Exons 17-20 | GCAGATCACCACCAGCAGAT |
| LYST X20-22F | Exons 20-22 | TGAAGGATGCTGTGGTGAAG |
| LYST X20-22R | Exons 20-22 | GTCCCTTATGAGCCCTGGAT |
| LYST X21-23F | Exons 21-23 | ATGCCCCGAGAGGTTTGTAG |
| LYST X21-23R | Exons 21-23 | AGTGCTGGGATTTCCAGTGG |
| LYST X23-26F | Exons 23-26 | AGTCCTGGGGATGAGTCCTG |
| LYST X23-26R | Exons 23-26 | CAGTAGCAGCCCTGAATGGATT |
| LYST X25-29F | Exons 25-29 | TGGTCGACATATTGGCCTTG |
| LYST X25-29R | Exons 25-29 | CGCAGAGACTACAGAGGCTC |
| LYST X28-32F | Exons 28-32 | AGCATTGCTGGTCCTCGAAA |
| LYST X28-32R | Exons 28-32 | TGGTCACAAGTGCATCCCTC |
| LYST X31F | Exon 31 | GCAACGGAGAATGAGCCAAG |
| LYST X31R | Exon 31 | CAGACGCTGAAAGAGACTTTGTT |
| LYST X31-35F | Exons 31-35 | CGCTGCACAAGAGAGAAAGC |
| LYST X31-35R | Exons 31-35 | GGGCCAAATCGAGAGAGGAG |
| LYST X34-39F | Exons 34-39 | GCAGTGTCTCTCTCCCAAGG |
| LYST X34-39R | Exons 34-39 | GCAGAACACTCCTGTTGGCA |
| LYST X37-42F | Exons 37-42 | TGCACCATCTAGAGAGACAGC |
| LYST X37-42R | Exons 37-42 | CGCAAAGGAGCCAGAGAAGA |
| LYST X40-45F | Exons 40-45 | TGCAGTATCCTGTGTTCCCA |
| LYST X40-45R | Exons 40-45 | GAGACGAGCGCTAGAAACCA |
| LYST X42-45F | Exons 42-45 | AAGAGTACCGCAAAGGAGCC |
| LYST X42-45R | Exons 42-45 | GGGTTGCTAGTGCAGTTTGC |
| LYST X43-44F | Exons 43-44 | GGAGCCAGAGAAGATGACCC |
| LYST X43-44R | Exons 43-44 | ATCCATGACAAAGGACTCGGAT |
| LYST X45-49F | Exons 45-49 | AAAACCTACGGGCAGACTCC |
| LYST X45-49R | Exons 45-49 | TGTGAGCAGAGACGGAACCT |
| LYST X48-53F | Exons 48-53 | CTGGAAGCAAATGCGGTGTC |
| LYST X48-53R | Exons 48-53 | TGGATGACTGGACAAACCATCC |

Abbreviations: cDNA, complementary DNA; F, forward; R, reverse.

**Supplementary Table 4.** Clinical summary of six individuals with Chediak-Higashi syndrome (CHS).

| **Individual** | **CHS subtype** | **Age at diagnosis** | **Pigment changes** | **Infections** | **Storage pool deficiency** | **Age at HSCT** | **Reference** |
| --- | --- | --- | --- | --- | --- | --- | --- |
| CHD3 | Classical | 7 years | Partial oculocutaneous albinism, silvery hair, pale skin, ocular albinism | Recurrent respiratory and skin infections | History of nosebleeds | 9 years | This study |
| CHD5 | Atypical | 19 years | Ocular albinism, decreased visual acuity | Cutaneous MRSA at 19 years | Gingival bleeding | N/A | (Gil-Krzewska et al., 2016; Introne et al., 2017) |
| CHD22 | Classical | 12 months | No ocular albinism; patchy hyper- and hypopigmentation of the skin | Omphalitis; recurrent sinopulmonary infections and otitis media | Platelet dense granule deficiency by EM | 1 year 8 months | This study |
| CHD25 | Classical | 3 years 6 months | Partial oculocutaneous albinism, silvery hair | Frequent bronchopulmonary infections | Unknown; low platelet count | N/A | This study |
| CHD34 | Classical | Neonatal | Partial oculocutaneous albinism; patchy hyper- and hypopigmentation of the skin | Pneumonia | Unknown | 8 months | This study |
| CHD36 | Classical | Neonatal | Partial oculocutaneous albinism | Thrush; delayed separation of umbilical cord, respiratory infections | Platelet dense granule deficiency by EM | 10 months | This study |

Abbreviations: CHS, Chediak-Higashi syndrome; EM, electron microscopy; HLH, hemophagocytic lymphohistiocytosis; HSCT, hematopoietic stem cell transplantation; MRSA, methicillin-resistant *Staphylococcus aureus*; N/A, not applicable.

**SUPPLEMENTARY REFERENCES**

Abou Tayoun, A.N., Pesaran, T., DiStefano, M.T., Oza, A., Rehm, H.L., Biesecker, L.G., et al. (2018). Recommendations for interpreting the loss of function PVS1 ACMG/AMP variant criterion. *Hum Mutat* 39(11)**,** 1517-1524. doi: <https://doi.org/10.1002/humu.23626>.

Biesecker, L.G., Harrison, S.M., and ClinGen Sequence Variant Interpretation Working, G. (2018). The ACMG/AMP reputable source criteria for the interpretation of sequence variants. *Genet Med* 20(12)**,** 1687-1688. doi: <https://doi.org/10.1038/gim.2018.42>.

Eckhart, L., Bach, J., Ban, J., and Tschachler, E. (2000). Melanin binds reversibly to thermostable DNA polymerase and inhibits its activity. *Biochem Biophys Res Commun* 271(3)**,** 726-730. doi: <https://doi.org/10.1006/bbrc.2000.2716>.

Giambernardi, T.A., Rodeck, U., and Klebe, R.J. (1998). Bovine serum albumin reverses inhibition of RT-PCR by melanin. *Biotechniques* 25(4)**,** 564-566. doi: <https://doi.org/10.2144/98254bm03>.

Gil-Krzewska, A., Wood, S.M., Murakami, Y., Nguyen, V., Chiang, S.C.C., Cullinane, A.R., et al. (2016). Chediak-Higashi syndrome: Lysosomal trafficking regulator domains regulate exocytosis of lytic granules but not cytokine secretion by natural killer cells. *J Allergy Clin Immunol* 137(4)**,** 1165-1177. doi: <https://doi.org/10.1016/j.jaci.2015.08.039>.

Introne, W.J., Westbroek, W., Groden, C.A., Bhambhani, V., Golas, G.A., Baker, E.H., et al. (2017). Neurologic involvement in patients with atypical Chediak-Higashi disease. *Neurology* 88(7)**,** e57-e65. doi: <https://doi.org/10.1212/WNL.0000000000003622>.

Livak, K.J., and Schmittgen, T.D. (2001). Analysis of relative gene expression data using real-time quantitative PCR and the 2(-Delta Delta C(T)) method. *Methods* 25(4)**,** 402-408. doi: <https://doi.org/10.1006/meth.2001.1262>.

Yarnell, D.S., Roney, J.C., Teixeira, C., Freitas, M.I., Cipriano, A., Leuschner, P., et al. (2020). Diagnosis of Chediak Higashi disease in a 67-year old woman. *Am J Med Genet A* 182(12)**,** 3007-3013. doi: <https://doi.org/10.1002/ajmg.a.61886>.
